# Supplementary material for: Exploring the Role of GMMA Components in the Immunogenicity of a 4-Valent Vaccine against Shigella
Source: Int J Mol Sci. 2023 Feb 1;24(3):2742. doi: 10.3390/ijms24032742 (PMC9916818; doi:10.3390/ijms24032742)
Supplement: Supplementary file 1 [file ijms-24-02742-s001.zip › ijms-2102030-supplementary.pdf]

Table S1. Most represented proteins on *S. flexneri* 1b OAg-positive and OAg-negative GMMA. CV: coefficient of variation. * t-test on difference between w/w values.

S. flexneri **1b GMMA**							
			**OAg⁻**		**OAg⁺**		
**Accession number**	**MW**	**Protein**	**% (w/w)**	**CV**	**% (w/w)**	**CV**	**Significance p-value***
WP_000865562.1	41402	porin OmpC [Enterobacteriaceae]	29.1	5%	33.3	1%	0.129
WP_005047463.1	37283	porin OmpA [Enterobacteriaceae]	32.8	6%	23.4	1%	0.086
WP_001295296.1	18603	outer membrane protein OmpX [Bacteria]	5.8	20%	4.9	7%	0.451
WP_000648420.1	8323	murein lipoprotein Lpp [Bacteria]	5.0	5%	3.5	41%	0.382
WP_000735285.1	53799	outer membrane channel protein TolC [Enterobacteriaceae]	4.2	4%	6.0	5%	**0.030**
WP_000597196.1	15602	outer membrane lipoprotein SlyB [Proteobacteria]	2.4	20%	2.3	25%	0.969
WP_000742443.1	49381	serine endoprotease DegP [Enterobacteriaceae]	2.4	4%	3.2	5%	0.045
WP_001295307.1	45956	Tol-Pal system protein TolB [Proteobacteria]	1.9	16%	1.6	2%	0.355
WP_000249126.1	72755	penicillin-binding protein activator LpoA [Enterobacteriaceae]	1.5	1%	0.6	116%	0.282
WP_001297320.1	36842	outer membrane protein assembly factor BamC [Proteobacteria]	1.4	4%	2.3	4%	**0.013**
WP_001240896.1	90553	outer membrane protein assembly factor BamA [Proteobacteria]	1.3	2%	1.8	3%	**0.027**
WP_000977934.1	39363	porin OmpF [Enterobacteriaceae]	1.2	5%	2.1	2%	**0.007**
WP_011069568.1	80957	ferric aerobactin receptor IutA [Enterobacteriaceae]	0.9	17%	1.0	9%	0.382
WP_001177043.1	41827	outer membrane protein assembly factor BamB [Enterobacteriaceae]	0.9	2%	1.5	5%	**0.045**
WP_000163771.1	27831	scaffolding protein MipA [Proteobacteria]	0.5	43%	0.6	16%	0.714
		**Tot (w/w)**	**91.4**		**88.1**		

Table S2. Most represented proteins on *S. flexneri* 2a OAg-positive and OAg-negative GMMA. CV: coefficient of variation, n.d.: not detected. * t-test on difference between w/w values.

S. flexneri **2a GMMA**							
			**OAg⁻**		**OAg⁺**		**Significance p-value***
**Accession number**	**MW**	**Protein**	**% (w/w)**	**CV**	**% (w/w)**	**CV**	
WP_000865562.1	41402	porin OmpC [Enterobacteriaceae]	30.5	14%	31.2	13%	0.887
WP_005047463.1	37283	porin OmpA [Enterobacteriaceae]	31.7	2%	16.1	2%	**0.006**
WP_000648420.1	8323	murein lipoprotein Lpp [Bacteria]	4.4	115%	7.4	8%	0.551
WP_000742443.1	49381	serine endoprotease DegP [Enterobacteriaceae]	1.3	3%	6.9	6%	**0.032**
WP_001295296.1	18603	outer membrane protein OmpX [Bacteria]	7.0	2%	3.9	6%	**0.010**
WP_001240896.1	90553	outer membrane protein assembly factor BamA [Proteobacteria]	0.8	116%	2.9	2%	0.195
WP_000597196.1	15602	outer membrane lipoprotein SlyB [Proteobacteria]	1.8	6%	2.6	29%	0.366
WP_000838272.1	28928	FKBP-type peptidyl-prolyl cis-trans isomerase [Enterobacteriaceae]	1.4	2%	2.1	6%	0.064
WP_000735285.1	53799	outer membrane channel protein TolC [Enterobacteriaceae]	0.9	5%	1.9	1%	**0.014**
WP_001240896.1	39363	porin OmpF [Enterobacteriaceae]	0.8	116%	2.9	2%	0.195
WP_001295307.1	45956	Tol-Pal system protein TolB [Proteobacteria]	0.7	114%	1.6	1%	0.387
WP_000249126.1	72755	penicillin-binding protein activator LpoA [Enterobacteriaceae]	1.2	0.2%	1.6	0.2%	**< 0.001**
WP_000800457.1	47284	peptidylprolyl isomerase SurA [Proteobacteria]	0.7	2%	1.3	4%	**0.037**
WP_001297320.1	36842	outer membrane protein assembly factor BamC [Proteobacteria]	0.6	1%	1.1	6%	0.064
WP_001177043.1	41827	outer membrane protein assembly factor BamB [Enterobacteriaceae]	0.5	2%	1.1	3%	**0.013**
WP_005049680.1	61019	oligopeptide ABC transporter substrate-binding protein OppA [Enterobacteriaceae]	0.6	4%	1.0	2%	**0.003**
		**Tot (w/w)**	**84.9**		**85.6**		

Table S3. Most represented proteins on *S. flexneri* 3a OAg-positive and OAg-negative GMMA. CV: coefficient of variation, nd: not detected. * t-test on difference among w/w values.

S. flexneri 3a GMMA							
			OAg⁻		OAg⁺		Significance p-value*
Accession number	MW	Description	% (w/w)	CV	% (w/w)	CV	
WP_000865562.1	41402	porin OmpC [Enterobacteriaceae]	33.0	3%	33.1	1%	0.970
WP_005047463.1	37283	porin OmpA [Enterobacteriaceae]	25.9	14%	22.8	2%	0.425
WP_000742443.1	49381	serine endoprotease DegP [Enterobacteriaceae]	2.2	18%	2.4	4%	0.689
WP_000648420.1	8323	murein lipoprotein Lpp [Bacteria]	7.0	29%	7.0	2%	0.988
WP_001295296.1	18603	outer membrane protein OmpX [Bacteria]	5.3	32%	3.4	0.4%	0.351
WP_001240896.1	90553	outer membrane protein assembly factor BamA [Proteobacteria]	2.6	24%	1.8	1%	0.338
WP_000597196.1	15602	outer membrane lipoprotein SlyB [Proteobacteria]	2.3	55%	2.9	1%	0.606
WP_000838272.1	28928	FKBP-type peptidyl-prolyl cis-trans isomerase [Enterobacteriaceae]	1.0	47%	1.2	5%	0.795
WP_001295307.1	45956	Tol-Pal system protein TolB [Proteobacteria]	1.1	42%	1.2	1%	0.696
WP_001177043.1	36842	outer membrane protein assembly factor BamC [Proteobacteria]	1.0	9%	0.8	12%	0.223
WP_001297320.1	41827	outer membrane protein assembly factor BamB [Enterobacteriaceae]	1.0	22%	0.9	0.3%	0.667
WP_000735285.1	53799	outer membrane channel protein TolC [Enterobacteriaceae]	2.7	3%	2.3	2%	**0.034**
WP_000800457.1	47284	peptidylprolyl isomerase SurA [Proteobacteria]	0.7	128%	0.6	6%	0.889
WP_001295306.1	18824	peptidoglycan-associated lipoprotein Pal [Proteobacteria]	0.6	120%	1.3	4%	0.382
WP_000768975.1	40893	glycerophosphodiester phosphodiesterase [Enterobacteriaceae]	0.7	98%	1.1	6%	0.497
WP_000737226.1	22928	outer membrane protein OmpW [Enterobacteriaceae]	1.3	65%	1.8	4%	0.559
		**Tot (w/w)**	**88.4**		**84.5**		

Table S4. Most represented proteins on *S. flexneri* OAg-positive GMMA. CV: coefficient of variation, nd: not detected. *One way Anova or t test.

			OAg⁺ GMMA						
			S. flexneri 1b		*S. flexneri* 2a		*S. flexneri* 3a		Significance p-value*
Accession number	MW	Description	% (w/w)	CV	% (w/w)	CV	% (w/w)	CV	
WP_000865562.1	41402	porin OmpC [Enterobacteriaceae]	33.3	1%	31.2	13%	33.1	1%	0.657
WP_005047463.1	37283	porin OmpA [Enterobacteriaceae]	23.4	1%	16.1	2%	22.8	2%	**< 0.001**
WP_001295296.1	18603	outer membrane protein OmpX [Bacteria]	4.9	7%	3.9	6%	3.4	0.4%	**0.019**
WP_000648420.1	8323	murein lipoprotein Lpp [Bacteria]	3.5	41%	7.4	8%	7.0	2%	**0.041**
WP_000735285.1	53799	outer membrane channel protein TolC [Enterobacteriaceae]	6.0	5%	1.9	1%	2.3	2%	**< 0.001**
WP_000597196.1	15602	outer membrane lipoprotein SlyB [Proteobacteria]	2.3	25%	2.6	29%	2.9	1%	0.621
WP_000742443.1	49381	serine endoprotease DegP [Enterobacteriaceae]	3.2	5%	6.9	6%	2.4	4%	**0.001**
WP_001295307.1	45956	Tol-Pal system protein TolB [Proteobacteria]	1.6	2%	1.6	1%	1.2	1%	**< 0.001**
WP_000249126.1	72755	penicillin-binding protein activator LpoA [Enterobacteriaceae]	0.6	116%	1.6	0.2%	nd	-	0.270
WP_001297320.1	36842	outer membrane protein assembly factor BamC [Proteobacteria]	2.3	4%	1.1	6%	0.9	0.3%	**< 0.001**
WP_001240896.1	90553	outer membrane protein assembly factor BamA [Proteobacteria]	1.8	3%	2.9	2%	1.8	1%	**< 0.001**
WP_000977934.1	39363	porin OmpF [Enterobacteriaceae]	2.1	2%	nd	-	nd	-	-
WP_011069568.1	80957	ferric aerobactin receptor IutA [Enterobacteriaceae]	1.0	9%	nd	-	nd	-	-
WP_001177043.1	41827	outer membrane protein assembly factor BamB [Enterobacteriaceae]	1.5	5%	1.1	3%	0.8	12%	**0.006**
WP_000163771.1	27831	scaffolding protein MipA [Proteobacteria]	0.6	16%	nd	-	nd	-	-
WP_000838272.1	28928	FKBP-type peptidyl-prolyl cis-trans isomerase [Enterobacteriaceae]	nd	-	2.1	6%	1.2	5%	**0.029**
WP_000800457.1	47284	peptidylprolyl isomerase SurA [Proteobacteria]	nd	-	1.3	4%	0.6	6%	**0.009**
WP_001295306.1	18824	peptidoglycan-associated lipoprotein Pal [Proteobacteria]	nd	-	nd	-	1.3	4%	-
WP_000768975.1	40893	glycerophosphodiester phosphodiesterase [Enterobacteriaceae]	nd	-	nd	-	1.1	6%	-
WP_000737226.1	22928	outer membrane protein OmpW [Enterobacteriaceae]	nd	-	nd	-	1.8	4%	-
WP_005049680.1	61019	oligopeptide ABC transporter substrate-binding protein OppA [Enterobacteriaceae]	nd	-	1.0	2%	nd	-	-
		**Total (w/w)**	**88.1**		**82.6**		**84.5**		

Table S5. Most represented proteins on *S. flexneri* OAg-negative GMMA. CV: coefficient of variation, nd: not detected. *One way Anova or t test.

OAg⁻ GMMA									
			***S. flexneri* 1b**		***S. flexneri* 2a**		***S. flexneri* 3a**		**Significance p-value***
**Accession number**	**MW**	**Description**	**% (w/w)**	**CV**	**% (w/w)**	**CV**	**% (w/w)**	**CV**	
WP_000865562.1	41402	porin OmpC [Enterobacteriaceae]	29.1	5%	30.5	14%	33.0	3%	0.448
WP_005047463.1	37283	porin OmpA [Enterobacteriaceae]	32.8	6%	31.7	2%	25.9	14%	0.115
WP_001295296.1	18603	outer membrane protein OmpX [Bacteria]	5.8	20%	7.0	2%	5.3	32%	0.466
WP_000648420.1	8323	murein lipoprotein Lpp [Bacteria]	5.0	5%	4.4	115%	7.0	29%	0.718
WP_000735285.1	53799	outer membrane channel protein TolC [Enterobacteriaceae]	4.2	4%	0.9	5%	2.7	3%	**< 0.001**
WP_000597196.1	15602	outer membrane lipoprotein SlyB [Proteobacteria]	2.4	20%	1.8	6%	2.3	55%	0.727
WP_000742443.1	49381	serine endoprotease DegP [Enterobacteriaceae]	2.4	4%	1.3	3%	2.2	18%	**0.040**
WP_001295307.1	45956	Tol-Pal system protein TolB [Proteobacteria]	1.9	16%	0.7	114%	1.1	42%	0.249
WP_000249126.1	72755	penicillin-binding protein activator LpoA [Enterobacteriaceae]	1.5	1%	1.2	0%	nd	-	**0.032**
WP_001297320.1	36842	outer membrane protein assembly factor BamC [Proteobacteria]	1.4	4%	0.6	1%	1.0	22%	**0.020**
WP_001240896.1	90553	outer membrane protein assembly factor BamA [Proteobacteria]	1.3	2%	0.8	116%	2.6	24%	0.151
WP_000977934.1	39363	porin OmpF [Enterobacteriaceae]	1.2	5%	nd	-	nd	-	-
WP_011069568.1	80957	ferric aerobactin receptor IutA [Enterobacteriaceae]	0.9	17%	nd	-	nd	-	-
WP_001177043.1	41827	outer membrane protein assembly factor BamB [Enterobacteriaceae]	0.9	2%	0.5	2%	1.0	9%	**0.005**
WP_000163771.1	27831	scaffolding protein MipA [Proteobacteria]	0.5	43%	nd	-	nd	-	-
WP_000838272.1	28928	FKBP-type peptidyl-prolyl cis-trans isomerase [Enterobacteriaceae]	nd	-	1.4	2%	1.0	47%	0.538
WP_000800457.1	47284	peptidylprolyl isomerase SurA [Proteobacteria]	nd	-	0.7	2%	0.7	128%	0.986
WP_005049680.1	61019	oligopeptide ABC transporter substrate-binding protein OppA [Enterobacteriaceae]	nd	-	0.6	4%	nd	-	-
WP_001295306.1	18824	peptidoglycan-associated lipoprotein Pal [Proteobacteria]	nd	-	nd	-	0.6	120%	-
WP_000768975.1	40893	glycerophosphodiester phosphodiesterase [Enterobacteriaceae]	nd	-	nd	-	0.7	98%	-
WP_000737226.1	22928	outer membrane protein OmpW [Enterobacteriaceae]	nd	-	nd	-	1.3	65%	-
		**Total (w/w)**	**91.4**		**84.1**		**88.4**		

Table S6. Composition of Chemically Defined Medium.

		Concentration			
**Reagent**	**Supplier Code#**	***S. sonnei***	***S flexneri* 1b**	***S flexneri* 2a**	***S flexneri* 3a**
Sulfuric Acid	VWR -1.00731.1000	[13]	20 μL/kg	20 μL/kg	20 μL/kg
Manganese chloride tetrahydrate	SIGMA -M8054		15 mg/kg	15 mg/kg	15 mg/kg
Copper Chloride dihydrate	SIGMA -307483		1.5 mg/kg	1.5 mg/kg	1.5 mg/kg
Boric Acid solution 4%	SIGMA-31144		75 mg/kg	75 mg/kg	75 mg/kg
Sodium Molybdate Dihydrate	SIGMA-M1651		2.5 mg/kg	2.5 mg/kg	2.5 mg/kg
Zinc acetate dihydrate	SIGMA -383058		13 mg/kg	13 mg/kg	13 mg/kg
Hexammine cobalt(III) chloride	SIGMA-H7891		2.5 mg/kg	2.5 mg/kg	2.5 mg/kg
Ferric Citrate	SIGMA -F3388		24.5 mg/kg	24.5 mg/kg	24.5 mg/kg
Magnesium Sulfate heptahydrate	SIGMA-63138		493.0 mg/kg	493.0 mg/kg	493.0 mg/kg
Thiamine Hydrochloride	SIGMA-T4562		50 mg/kg	50 mg/kg	50 mg/kg
NIcotinic Acid	SIGMA -N0761		10 mg/kg	10 mg/kg	10 mg/kg
Potassium Phosphate Monobasic	SIGMA-P5655		13.3 g/kg	13.3 g/kg	13.3 g/kg
Di-Ammonium HydrogenPhosphate	SIGMA-215996		4 g/kg	4 g/kg	4 g/kg
Citric Acid Monohydrate	SIGMA-C1909		1.7 g/kg	1.7 g/kg	1.7 g/kg
Dextrose monohydrate	SIGMA -D9559		5 g/kg	5 g/kg	5 g/kg
L-Aspartic acid	SIGMA -A7219		2.5 g/kg	2.5 g/kg	2.5 g/kg
L-Methionine	SIGMA-M5308		0.16 g/kg	0 g/kg	0.16 g/kg

L-Asparagine	SIGMA-A4159		0.06 g/kg	0 g/kg	0.06 g/kg
L-Threonine	SIGMA-T8441		0.22 g/kg	0 g/kg	0.22 g/kg
L-Lysine monohydrate	SIGMA-L5510		0.60 g/kg	0 g/kg	0.67 g/kg
L-Glutamic acid monosodium salt monohydrate	SIGMA-49621		1.78 g/kg	0 g/kg	1.78 g/kg

Table S7. HPLC-RP gradient. Solvent A: 5% Acetonitrile, 15% Isopropyl alcohol, 0.3% TFA in water (by volume); Solvent B: 80% Acetonitrile, 15% Isopropyl alcohol, 0.3% TFA in water (by volume); Solvent C: Isopropyl alcohol. Column temperature: 70° C.

Time	Flow (mL/min)	%A	%B	%C	Curve
Initial	0.2	100	0	0	Initial
2	0.2	100	0	0	6
8	0.2	70	30	0	6
13	0.2	55	45	0	6
24	0.2	0	100	0	6
28	0.2	0	100	0	6
34	0.2	0	0	100	6
44	0.2	0	0	100	6
49	0.2	100	0	0	6
69	0.2	100	0	0	6

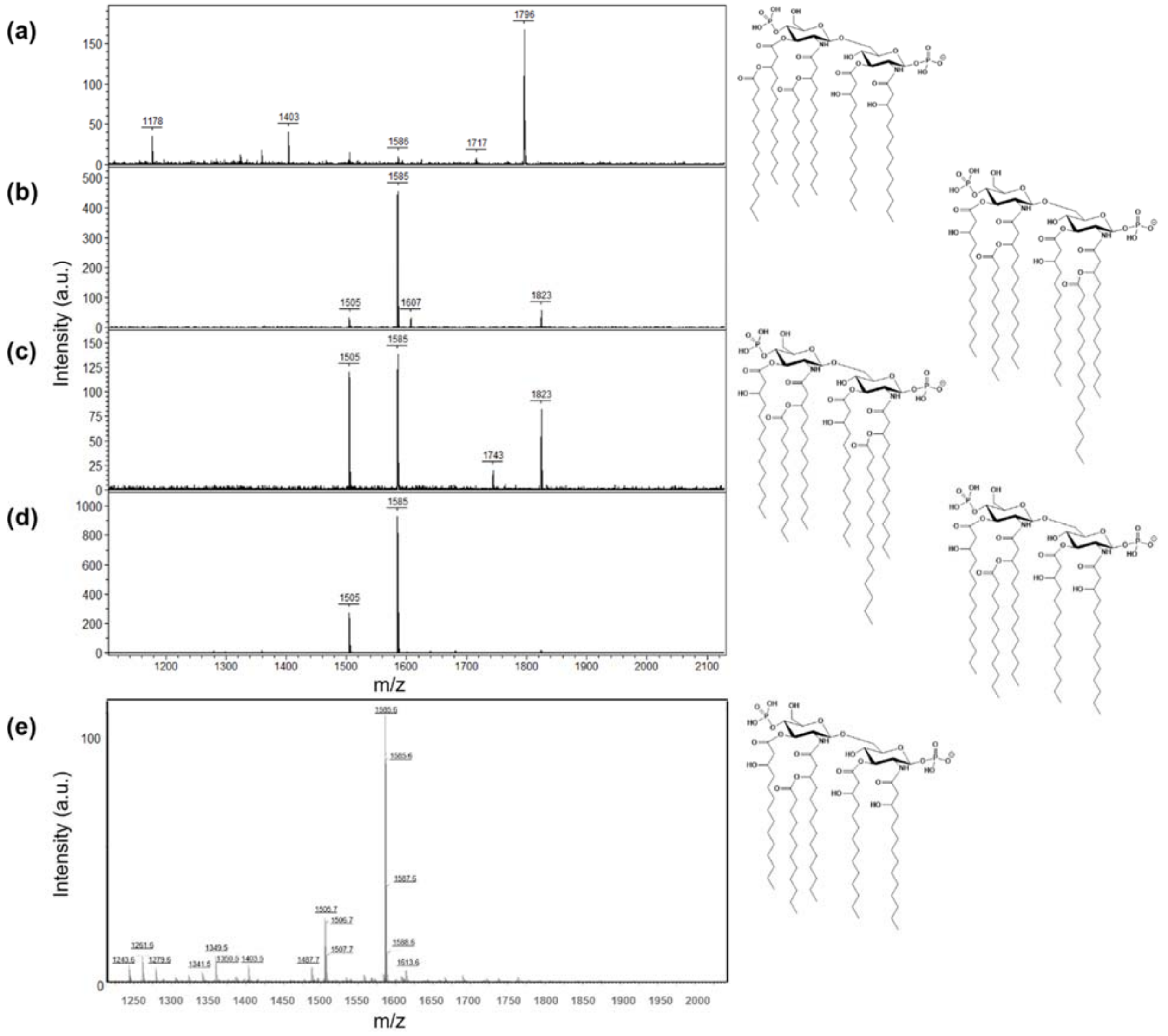

Figure S1. MALDI-TOF spectra and structures of lipidA. (a) wild type lipid A, (b) *S. flexneri* 1b GMMA lipid A, (c) *S. flexneri* 2a GMMA lipid A, (d) *S. flexneri* 3a GMMA lipid A, (e) S. sonnei GMMA lipid A.

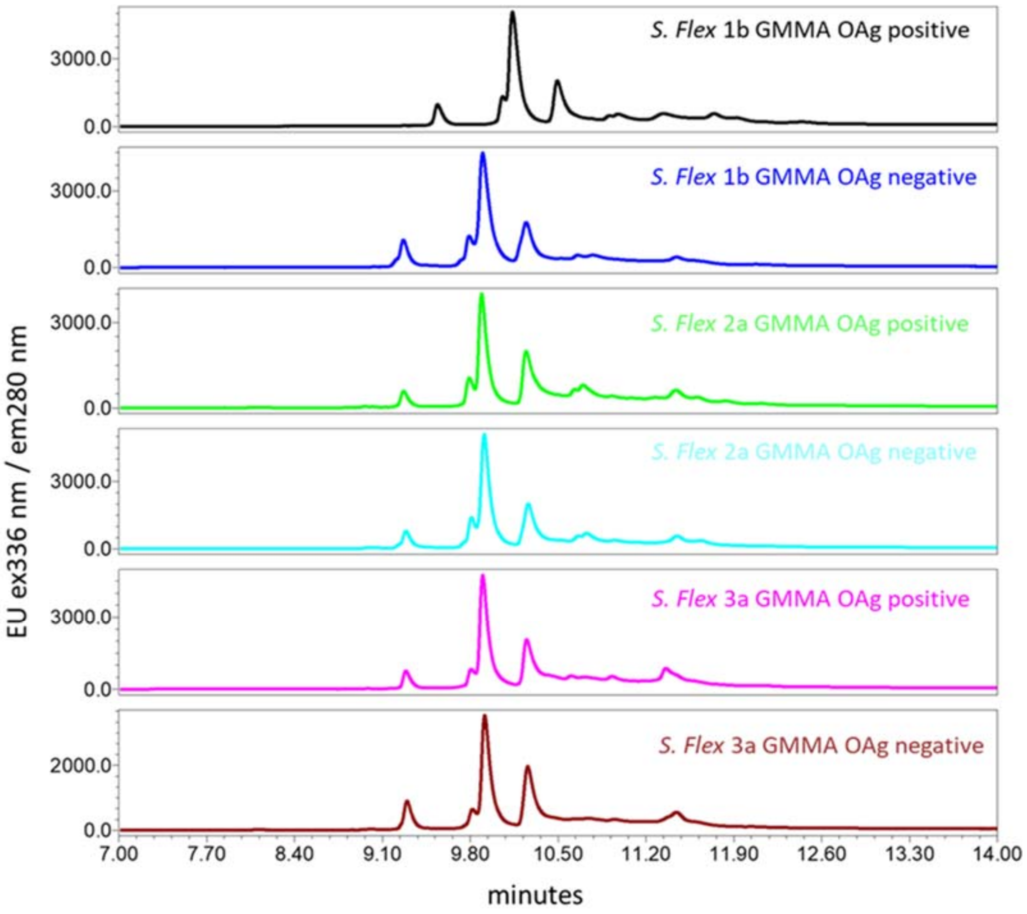

Figure S2. GMMA HPLC-RP protein profile.
